# A Full-Length *Plasmodium falciparum* Recombinant Circumsporozoite Protein Expressed by *Pseudomonas fluorescens* Platform as a Malaria Vaccine Candidate

**DOI:** 10.1371/journal.pone.0107764

**Published:** 2014-09-23

**Authors:** Amy R. Noe, Diego Espinosa, Xiangming Li, Jordana G. A. Coelho-dos-Reis, Ryota Funakoshi, Steve Giardina, Hongfan Jin, Diane M. Retallack, Ryan Haverstock, Jeffrey R. Allen, Thomas S. Vedvick, Christopher B. Fox, Steven G. Reed, Ramses Ayala, Brian Roberts, Scott B. Winram, John Sacci, Moriya Tsuji, Fidel Zavala, Gabriel M. Gutierrez

**Affiliations:** 1 Leidos Inc., Frederick, Maryland, United States of America; 2 Johns Hopkins Malaria Research Institute and Department of Molecular Microbiology and Immunology, Johns Hopkins Bloomberg School of Public Health, Johns Hopkins University, Baltimore, Maryland, United States of America; 3 HIV and Malaria Vaccine Program, Aaron Diamond AIDS Research Center, Affiliate of The Rockefeller University, New York, New York, United States of America; 4 Pfenex Inc., San Diego, California, United States of America; 5 Infectious Disease Research Institute, Seattle, Washington, United States of America; 6 Department of Microbiology and Immunology, University of Maryland School of Medicine, Baltimore, Maryland, United States of America; Ehime University, Japan

## Abstract

The circumsporozoite protein (CSP) of *Plasmodium falciparum* is a major surface protein, which forms a dense coat on the sporozoite's surface. Preclinical research on CSP and clinical evaluation of a CSP fragment-based RTS, S/AS01 vaccine have demonstrated a modest degree of protection against *P. falciparum*, mediated in part by humoral immunity and in part by cell-mediated immunity. Given the partial protective efficacy of the RTS, S/AS01 vaccine in a recent Phase 3 trial, further improvement of CSP-based vaccines is crucial. In this report, we describe the preclinical development of a full-length, recombinant CSP (rCSP)-based vaccine candidate against *P. falciparum* malaria suitable for current Good Manufacturing Practice (cGMP) production. Utilizing a novel high-throughput *Pseudomonas fluorescens* expression platform, we demonstrated greater efficacy of full-length rCSP as compared to N-terminally truncated versions, rapidly down-selected a promising lead vaccine candidate, and developed a high-yield purification process to express immunologically active, intact antigen for clinical trial material production. The rCSP, when formulated with various adjuvants, induced antigen-specific antibody responses as measured by enzyme-linked immunosorbent assay (ELISA) and immunofluorescence assay (IFA), as well as CD4+ T-cell responses as determined by ELISpot. The adjuvanted rCSP vaccine conferred protection in mice when challenged with transgenic *P. berghei* sporozoites containing the *P. falciparum* repeat region of CSP. Furthermore, heterologous prime/boost regimens with adjuvanted rCSP and an adenovirus type 35-vectored CSP (Ad35CS) showed modest improvements in eliciting CSP-specific T-cell responses and anti-malarial protection, depending on the order of vaccine delivery. Collectively, these data support the importance of further clinical development of adjuvanted rCSP, either as a stand-alone product or as one of the components in a heterologous prime/boost strategy, ultimately acting as an effective vaccine candidate for the mitigation of *P. falciparum*-induced malaria.

## Introduction

World-wide, mortality from malaria is estimated in the hundreds of thousands each year, with more than 200 million cases estimated for 2012 [1]. Given the incidence of this disease, development of a malaria vaccine is a priority [2]. However, the complex lifecycle of the malaria parasite has, in part, hampered efforts to achieve this goal. Briefly, parasites are first delivered into the human host as sporozoites by the bite of an infected mosquito, and then rapidly travel to the liver where they invade hepatocytes. Once in the liver, they develop into merozoites and are subsequently released into the blood stream, where they invade red blood cells. Merozoite multiplication results in many thousands of parasite-infected erythrocytes in the host bloodstream, leading to clinical disease. Therefore, targeting a vaccine that prevents the parasite from entering and/or exiting the liver would provide the most effective prevention of disease. For children younger than age 5 residing in endemic regions of Africa and other developing nations, sterile immunity is the most desirable outcome of a vaccine [1]. The strategy of immunizing with high doses of irradiated sporozoites has, in fact, achieved immunity to the pre-erythrocytic stages of *Plasmodium* [3], [4]. Importantly, immune response to circumsporozoite protein (CSP) has been shown to be a necessary component of the protective immune response induced by irradiated sporozoite, since in the absence of an immune response to CSP, protection in mice achieved upon vaccination with irradiated sporozoites was greatly diminished compared to that in mice mounting an active immune response to CSP [5], [6].

CSP is the major surface protein of the sporozoite and forms a dense coat on the parasite's surface. Structurally, CSP is divided into three regions: (1) the NH_2_-terminal region, which includes the ligand-binding domain [7], multiple human HLA-restricted epitopes [8], [9], a proteolytic processing site [10], and a region I; (2) a centrally-located repeat region; and (3) the COOH-terminus, which contains a type I thrombospondin repeat region. Since its discovery more than 30 years ago, much attention has been given to developing a vaccine against CSP [11]. Antibody response to sporozoites is largely to the immuno-dominant central repeat region of CSP, and previous studies have shown that high titers of antibodies to this region can confer protection from malaria infection in rodent models [12], [13], [14]; however, translating these results to humans has been difficult. Various CSP configurations have only generated a sufficient immune response to confer partial protection, and the precise specificity and function of protective antibodies has not been determined. Considerable evidence from studies in animals and humans suggests that both antibody and T-cell immune responses are likely needed to control malaria infection [15], [16], [17], [18], [19], [20], [21], [22], [23].

Phase 2 clinical trials of an RTS, S/AS01 vaccine, a virus-like particle (VLP) consisting of a fragment (central repeat and C-terminal regions) of the CSP fused to a Hepatitis B Virus Surface Antigen (HBVsAg) (developed by GlaxoSmithKline), provided evidence for a high level of anti-CSP antibody response that correlated with reduced clinical malaria episodes. However, analysis of the Phase 3 trial of the RTS, S/AS01 vaccine showed that only one third of infants were protected by the CSP-fragment-based vaccine candidate [24]. The partial protective efficacy of the RTS, S/AS01 vaccine has demonstrated viability of a CSP-based malaria vaccine and suggests that further improvement of a CSP-based vaccine may provide an increase in clinical efficacy. Considering the nature of domains contained within the N-terminus of the protein, one key approach by which to improve the efficacy of a CSP-based subunit vaccine would be to utilize the full-length protein. In fact, data indicate that antibody responses to the N-terminus of CSP are associated with protection [25], [26]. Therefore, it is reasonable to postulate that a properly folded, full-length CSP will enhance the quality, magnitude, and breadth of protective antibody and T-cell responses. Indeed, a recent report showed that a full-length, recombinant *P. falciparum* CSP, expressed in *E. coli*, provided significant protection when administered with adjuvants in a malaria mouse challenge model [27]. However, manufacture of adequate amounts of clinical-grade full-length rCSP has been problematic [28]. Specifically, current Good Manufacturing Practice (cGMP) production of the protein at a large economical scale suitable for clinical evaluation and advanced product development has yet to be reported. This lack of clinical grade, full-length CSP may be due in large part to inherent unique properties of the *P. falciparum* parasite, which include an extremely A/T-rich genome with many lysine and arginine repeats, and proteins that contain multiple disulfide bonds. Expression of malaria proteins in bacterial systems, such as *E. coli*, often results in insoluble expression that requires purification from inclusion bodies and steps to refold the protein. Furthermore, malaria parasites lack N-linked glycosylation machinery, thereby making common eukaryotic expression platforms less effective. Therefore, as part of our CSP vaccine development strategy, we sought novel expression platforms to overcome the inherent obstacles associated with expression of malaria antigens listed above.

Here, we describe the successful expression and pre-clinical testing of a recombinant, full-length *P. falciparum* CSP that is immunogenic and biologically active. Briefly, we employed a high-throughput process to rapidly screen hundreds of *Pseudomonas fluorescens* expression strains starting from plasmid construction with varying promoters, secretion leaders, and translation initiation sequences. Strains producing soluble, high-yield, and full-length *P. falciparum* rCSP were identified and characterized. The purified full-length recombinant CSP (rCSP) was tested in animal studies for immunogenicity and efficacy in combination with several adjuvant formulations. Initially, incomplete Freund's adjuvant was used to establish baseline activity across a panel of biological assays. Other adjuvants more appropriate for use in humans were later evaluated with rCSP, including Alhydrogel, AdjuPhos, Glucopyranosyl Lipid Adjuvant-Stable Emulsion (GLA-SE), and a GLA-liposome-QS21 formulation (GLA-LSQ). When adjuvanted with GLA formulations, rCSP produced robust antibody titers that neutralized invasion of malarial sporozoites into hepatocytes *in vitro*. Furthermore, immunization with GLA rCSP formulations was effective at inhibiting liver stage development in mice (>90%) using a transgenic parasite model of infection. Although a detectable CD4+ T-cell response was seen with GLA formulations in mice, CD8+ T-cell response was low. In an attempt to improve the magnitude of T-cell response, a heterologous prime-boost strategy with rCSP and Ad35CS was employed. Ad35CS was used because of its low seroprevalence in endemic regions and its safety profile in the clinic [29]. Interestingly, we found that the order in which the various components of the prime-boost strategy were administered played a significant role in eliciting immunologically potent effects.

This study corroborates the hypothesis that immunization with full-length CSP can effectively induce both humoral and cell-mediated immune responses. Such a vaccine is more likely to achieve sufficient levels of sterilizing immunity by antibody neutralization of sporozoites prior to hepatocyte infection and/or through T-cell-mediated clearing of malaria-infected hepatocytes. Collectively, this is the first report of a process for generating clinical-grade full-length recombinant CSP that is not only manufacturable at a robust scale, but also is highly immunogenic, thus allowing us to swiftly move the rCSP forward for clinical evaluation.

## Results

### Design and Selection of rCSP Expression Strains

**Figure 1:**
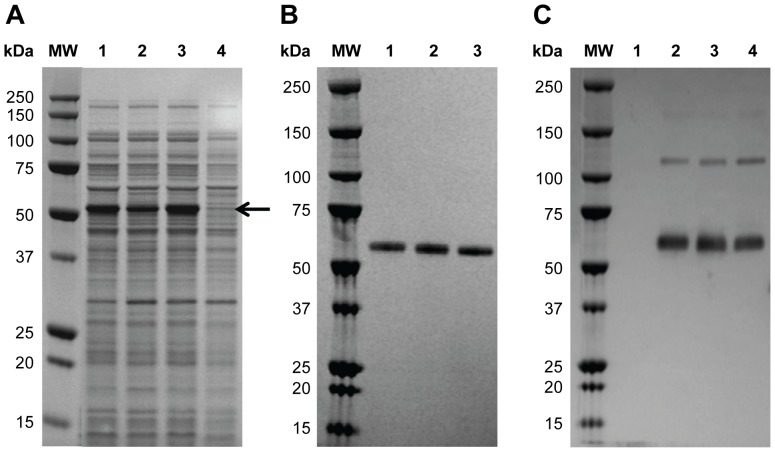
rCSP Strain Selection and Identification. (**A**) Reduced SDS-PAGE analysis of lysate samples prior to purification. Lanes 1–3 represent samples from strain CSP-1, CSP-3, and CSP-2 fermentations, respectively. Lane 4 represents a sample from a culture of *P. fluorescens* transformed with plasmid vector containing no insert. Arrow denotes rCSP in lysate samples. (**B**) Reduced SDS-PAGE analysis of purified proteins from a 2-column purification process. Lanes 1–3 represent samples from CSP-1, CSP-2, and CSP-3 productions, respectively. (**C**) Western blot of non-reduced purified CSP-1, CSP-2, and CSP-3 proteins (lanes 2–4, respectively). Lane 1 represents a sample from a culture of *P. fluorescens* transformed with plasmid vector containing no insert.

Twenty *P. fluorescens* host strains, including protease deletion mutants and strains expressing protein folding modulators, were each transformed with sixteen different expression plasmids comprised of varying secretion leader sequences and ribosome binding site combinations. The resulting 320 *P. fluorescens* expression strains were screened for rCSP expression at the 0.5 mL scale. Five strains with the highest level of expression were selected from the initial screening and evaluated at the 4 mL fermentation scale, under 9 different fermentation conditions such as varied combinations of inducer concentration, induction pH, and temperature (data not shown). Three strains expressing high levels of rCSP (CSP-1, CSP-2, and CSP-3) were selected for further characterization (Figure 1a). Conditions were then scaled up to a high cell density fermentation process in 1 L conventional bioreactors where yields ranged up to 4 g/L depending on strain and fermentation condition tested (data not shown). Purification was initially performed using a 2-column method with anion-exchange and hydrophobic interaction chromatography to achieve material of sufficient purity (>90%) for analytical assessment and in vivo functional tests. SDS-PAGE analysis showed a single protein band run at apparent molecular weight ∼55 kDa for all three strains (Figure 1b). Western blot analysis using an anti-CSP mAb confirmed the band as CSP (Figure 1c). A faint band at approximately 120 kDa was also visible by western blot and represents a dimeric form of rCSP not reduced under the sample preparation conditions used for the western blot assay.

### Purification and Analytical Assessment of rCSP

**Figure 2:**
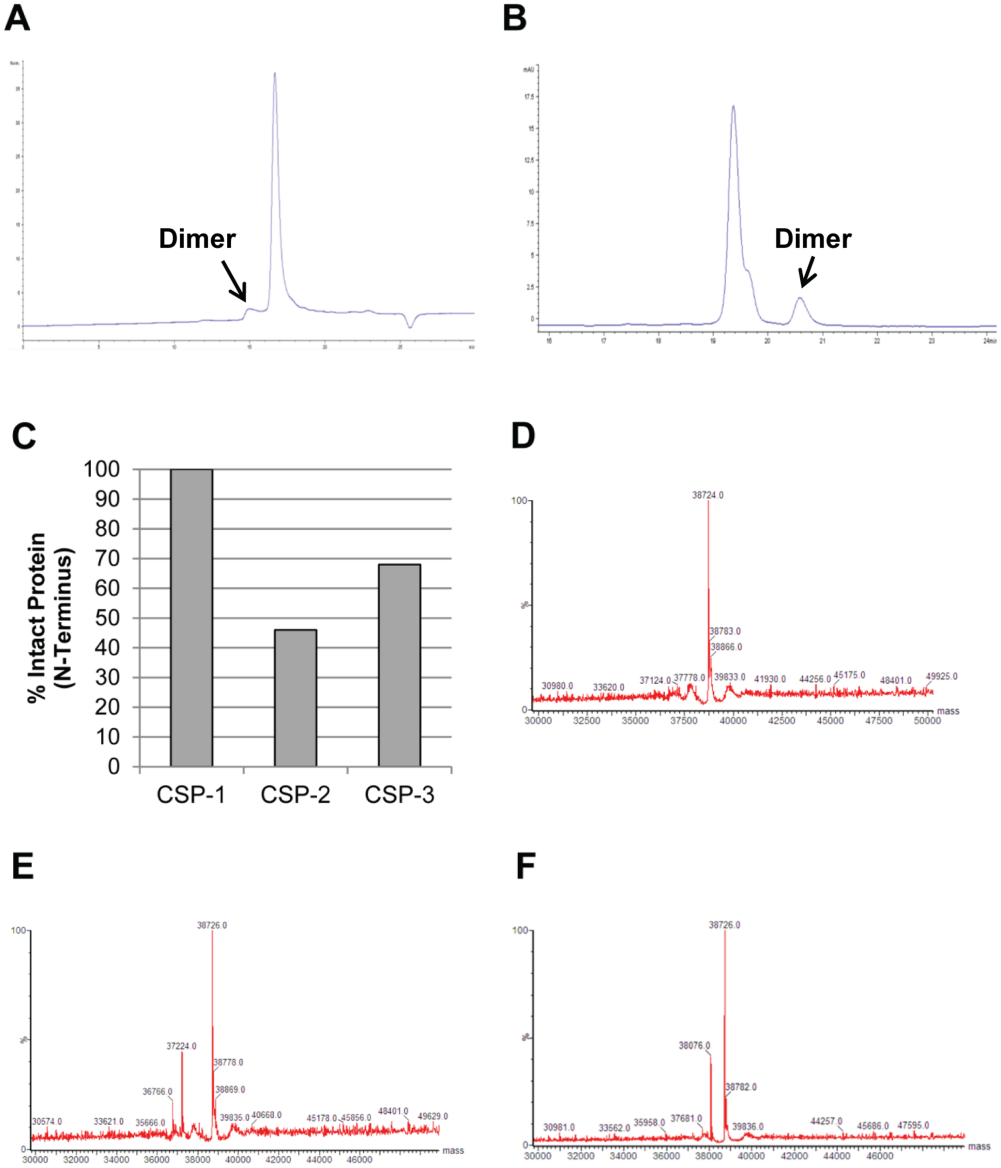
Analytical Assessment of rCSP Purity. Expression products from the three strains (CSP-1, CSP-2, and CSP-3) were characterized using a panel of analytical assays. (**A**) A SE-HPLC chromatogram of rCSP from the CSP-1 strain purified with the 2-column process shows a major peak representing monomeric rCSP and minor peak representing dimeric rCSP. The dimer represents ∼9% of the rCSP present. (**B**) A RP-HPLC chromatogram of rCSP from the CSP-1 strain purified with the 2-column process shows a major peak representing monomeric rCSP and minor peak representing dimeric rCSP. The dimer represents ∼9.5% of the rCSP present. (**C**) N-terminal sequencing demonstrated that the CSP-1 protein was fully intact; however, CSP-2 and CSP-3 showed clipping at the N-terminus. (**D**) Intact mass analysis of rCSP from the CSP-1 strain showed a single peak at 38724 Da. (**E**) Intact mass analysis of rCSP from the CSP-2 strain showed one peak at 38,726 Da and two truncated forms with minor peaks at 37,224 Da and 36,766 Da. (**F**) Intact mass analysis of rCSP from the CSP-3 strain showed one peak at 38726 Da and one truncated form with a minor peak at 38,076 Da.

In addition to the initial assessments by gel electrophoresis, the purity and the extent of rCSP multimerization for all lots were analyzed by size exclusion (SE) and reversed phase (RP) high-performance liquid chromatography (HPLC). During development of the purification process, a strong tendency of the protein to multimerize was alleviated by addition of a disaggregant (urea) and a reducing agent to process buffers. A representative SE-HPLC chromatogram shows a minor peak at approximately 15 minutes corresponding to the rCSP dimer, and a major peak at approximately 17 minutes corresponding to the rCSP monomer (Figure 2a). A representative RP-HPLC chromatogram shows a major peak at approximately 19.2 minutes corresponding to the rCSP monomer with a detectable shoulder that was identified as a pyroglutamic acid derivative of rCSP (data not shown) as well as a minor peak at approximately 20.6 minutes corresponding to the rCSP dimer (Figure 2b). Based on HPLC results, the percentage of dimer was estimated as <10% after the 2-column purification process and final buffer exchange.

To confirm identity and integrity of the expression products, N-terminal sequencing and intact mass analysis were performed. Sequencing the N-terminus of each expression product confirmed the proteins as CSP for all three strains. In addition, it was determined that rCSP from strain CSP-1 was 100% intact, rCSP from strain CSP-2 was only 46% intact with clipping occurring after amino acid 13 or amino acid 17, and rCSP from strain CSP-3 was 68% intact with the first 5 amino acids being trimmed from the clipped protein (Figure 2c). Intact mass analysis confirmed these results as the transformed and integrated maximum entropy spectra revealed the presence of a principal mass component at 38,724–38,726 Da for all three samples; the theoretical molecular weight for full-length CSP matches at 38,725 Da. A single full-length expression product was found for strain CSP-1 (Figure 2d), while the other two strains had N-terminal truncated variants present. For the CSP-2 strain, two truncated rCSP species were found, one starting at Val14 and the other at Leu18 with actual molecular weights of 36,766 Da and 37,224 Da, and theoretical molecular weights of 37,220 Da and 36,765 Da, respectively (Figure 2e). For the CSP-3 strain, one truncated species was found starting at Tyr6 with an actual molecular weight of 38,076 Da and a theoretical molecular weight of 38,073 Da (Figure 2f).

### Biological and Functional Evaluation of rCSP Strains

**Figure 3:**
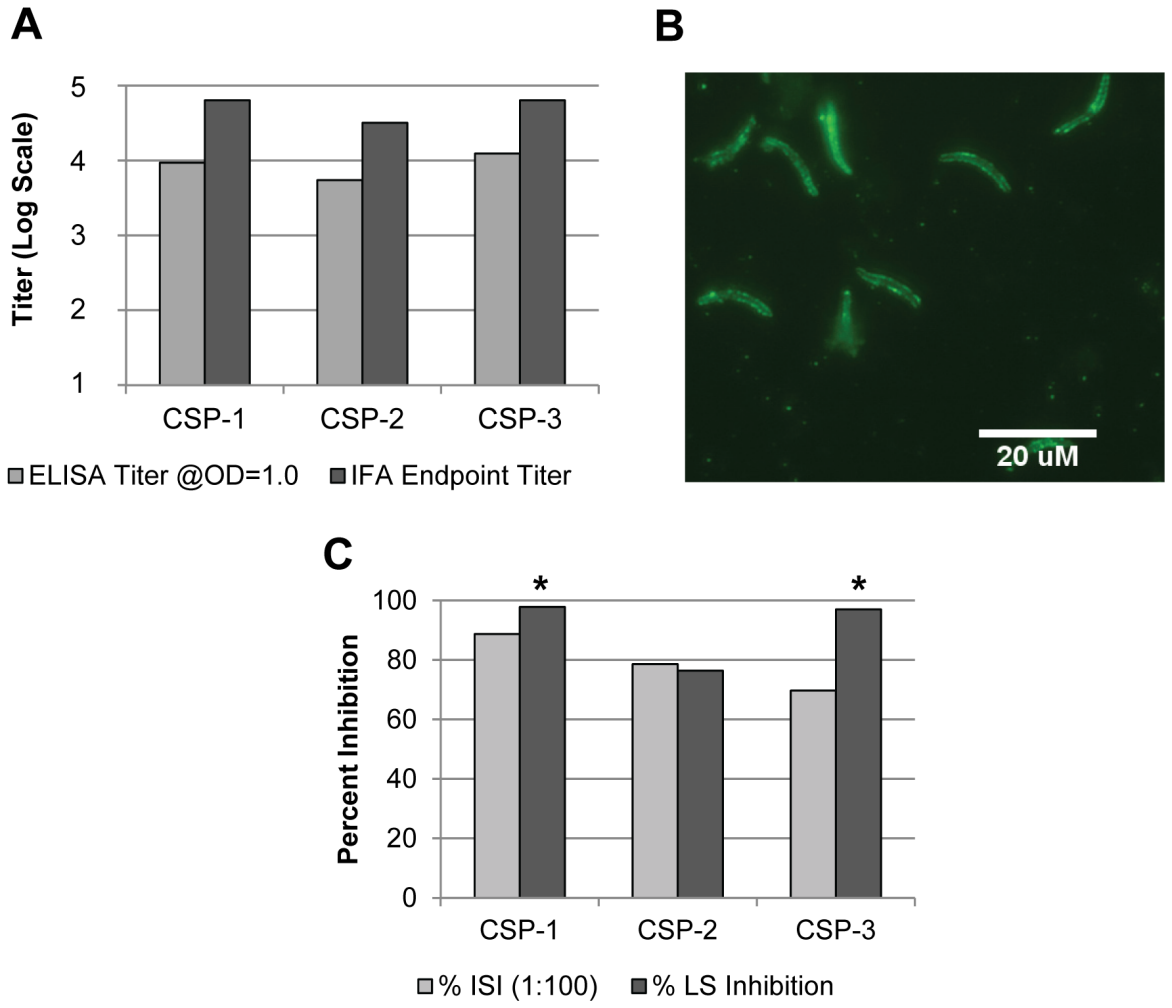
Immunogenicity and Efficacy of rCSP Immunization in Mice. Five C57BL/6 mice per cohort were immunized 3x with 25 µg of rCSP from 3 production strains (CSP-1, CSP-2, and CSP-3) formulated in incomplete Freund's adjuvant. Sera were collected 2 weeks after the last immunization and pooled for analysis by ELISA, IFA, and ISI. Mice were challenged with *Pb*-CS(*Pf*) parasites 3 weeks after the last immunization. (**A**) Pooled sera from mice immunized with the 3 recombinant proteins showed similar ELISA and IFA titers. ELISA titers to a [NANP]_6_ peptide were calculated at OD = 1.0 based on 4-parameter logistic curve fits, and IFA titers were determined based on the lowest serum dilution to give sporozoites-specific fluorescence above background level shown by sera from naïve mice. (**B**) Sera from rCSP-immunized mice reacted to *Pb*-CS(*Pf*) sporozoites demonstrated the expected pattern of surface fluorescence by IFA. (**C**) Sera from rCSP-immunized mice diluted 1∶100 and assayed for the ability to block *Pf* sporozoite infection of hepatocytes in an ISI assay demonstrated the highest ISI activity for anti-CSP-1 protein sera. The percent inhibition is shown relative to the number of sporozoites after incubating with the sera of naïve mice. Percent inhibition of LS parasite development in rCSP-immunized mice challenged with *Pb*-CS(*Pf*) sporozoites normalized to naïve control mice is also shown. The reduction of LS parasites in livers of mice immunized with CSP-1 or CSP-3 proteins was statistically significant based on the mean parasite-specific 18 s rRNA copy number compared the level of LS parasites in livers of naïve and adjuvant-only administered mice (as noted by asterisks).

To address whether the CSP strains have biological activity and whether the N-terminus of the CSP protein impacts its biological function, we tested all three expression products for their ability to elicit an immune response in vivo. Furthermore, the ability of the CSP strains to induce protective anti-plasmodial response was assessed by quantifying the in vitro parasite neutralization activity of the generated antisera and the in vivo inhibition of liver stage development in a mouse model. For this initial study, CSP-1, CSP-2, and CSP-3 derived proteins were formulated in incomplete Freund's adjuvant. ELISA titers were similar among pooled sera from mice immunized with protein generated from the three strains, as measured against a CSP repeat region peptide [NANP]_6_ by ELISA (Figure 3a). Optical density (OD) in sera from mice injected only with incomplete Freund's adjuvant (the adjuvant control) was less than 0.1. In addition to ELISA, immunogenicity was also measured via immunofluorescence assay IFA using transgenic *P. berghei* sporozoites containing the *P. falciparum* repeat region and portion of the N-terminal region of CSP (*Pb-CS[Pf]*) (Figure 3a). As with ELISA, the IFA titers were similar among sera from mice immunized with the three recombinant proteins. The IFA also confirmed reactivity of all the anti-rCSP sera to the surface of *Pb*-CS(*Pf*) sporozoites (Figure 3b, only reactivity of anti-CSP-1 sera is shown). There was no specific sporozoite surface fluorescence seen with the sera from the adjuvant control mouse cohort at the highest concentration tested (data not shown). To determine whether ELISA and IFA titers were associated with functional antibodies, the same sera pools were assessed in an inhibition of sporozoite invasion (ISI) assay with a cultured hepatocytoma cell line. More pronounced differences among the three sera pools were seen in the ISI assay (Figure 3c). Indeed, a 1∶100 dilution of anti-CSP-1 serum achieved an 89% reduction in the level of hepatocyte invasion by *P. falciparum* sporozoites (as compared to naïve mouse sera); however, similar dilutions of anti-CSP-2 or anti-CSP-3 sera were only able to attain 77% and 70% invasion reduction, respectively. To further compare protective efficacy of the three rCSP expression products, immunized mice were challenged with *Pb*-CS(*Pf*) sporozoites to determine the degree of the inhibition of liver stage (LS) parasite development (Figure 3c). The reduction in parasite liver load in mice immunized with CSP-1 or CSP-3 proteins was statistically significant compared to naïve mice and mice administered adjuvant alone. However, this was not the case with mice immunized with CSP-2. The reduction in liver load in mice administered CSP-1, CSP-2, or CSP-3 proteins was 98% (p = 0.008), 76% (p = 0.151), and 97% (p = 0.008), respectively, when standardized to the naïve mouse control group. Liver parasite load in mice administered adjuvant alone was similar to that of the naïve control group with only a non-significant decrease (16%) seen in the adjuvant alone group as compared to the naïve control group. The results from the ISI and LS inhibition assays suggest that even small deletions at the N terminal can have a deleterious effect on immunogenic efficacy; therefore, based on the percentage of intact sequence and the optimal performance in the protective efficacy assays, CSP-1 was selected as the lead candidate strain for further rCSP production and rCSP-based vaccine development.

### Adjuvant Formulation Screening for rCSP Vaccine

**Figure 4:**
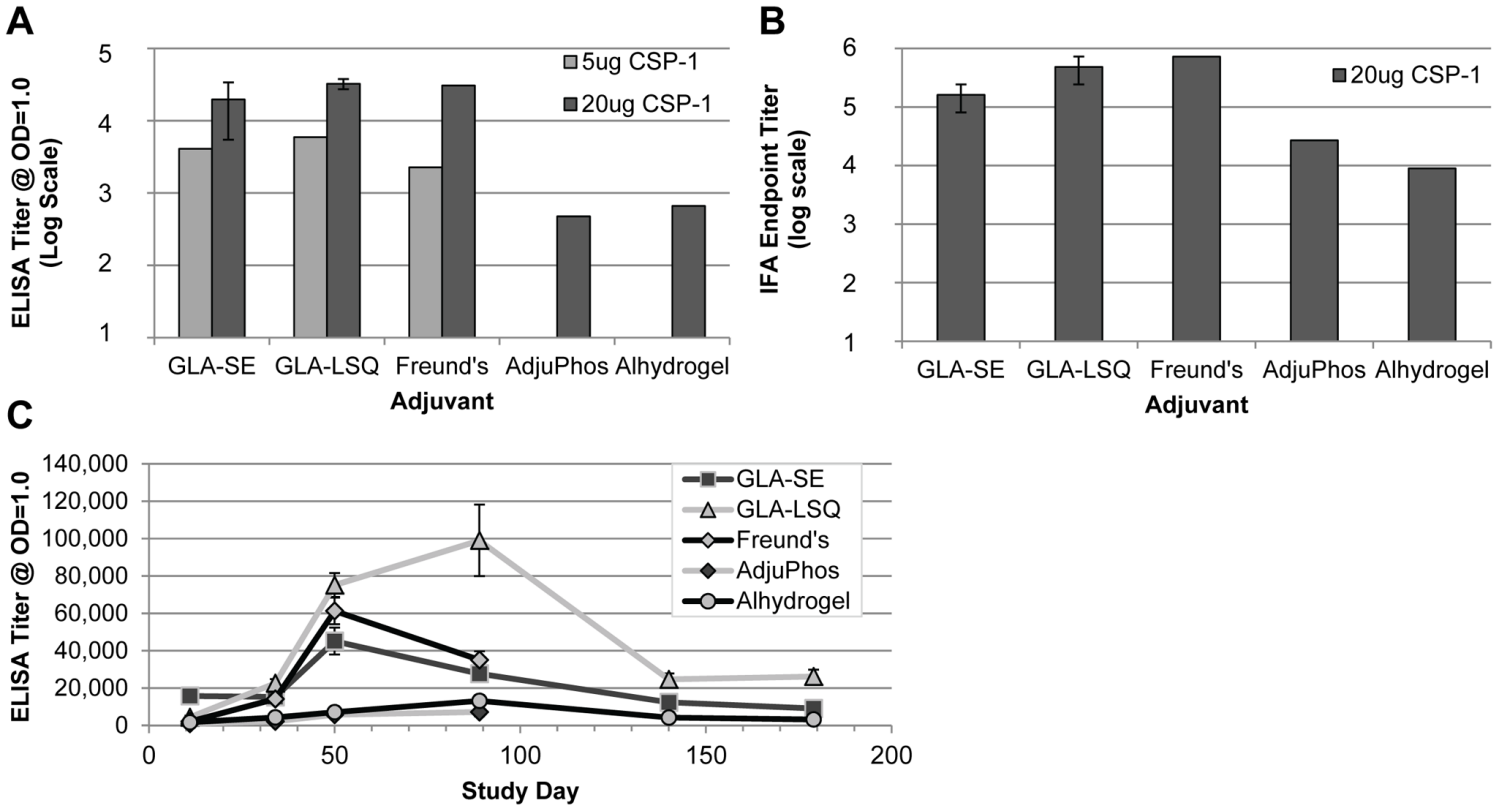
Comparison of Humoral Responses with rCSP Adjuvant Formulations in Mice. C57BL/6 mice were immunized 3x with 2.5 µg, 5 µg, or 20 µg of rCSP (CSP-1) formulated in different adjuvants. (**A**) Pooled sera (7 mice per cohort) collected 2 weeks after the final immunization from mice immunized with GLA-SE, GLA-LSQ and incomplete Freund's adjuvant showed similar ELISA titers to a [NANP]_6_ peptide at OD = 1.0 based on 4-parameter logistic curve fits. The titers of the sera from mice injected with rCSP formulated in alum-based adjuvants were lower. The immunogenicity of GLA-SE and GLA-LSQ formulations with 20 µg rCSP was assessed in multiple independent studies (5–7 mice per cohort were used for each study); average titer is shown with error bars represented as standard error. (**B**) Pooled sera (7 mice per cohort) collected 2 weeks after the final immunization from mice immunized with GLA-SE, GLA-LSQ and incomplete Freund's adjuvant demonstrated the highest IFA endpoint titers based on the lowest serum dilution to give *Pb*-CS(*Pf*) sporozoite surface fluorescence above negative sera control level. The titers of the sera from mice immunized with rCSP formulated in alum-based adjuvants were lower. The immunogenicity of GLA-SE and GLA-LSQ formulations with 20 µg rCSP were assessed in multiple independent studies (5–7 mice per cohort were used for each study); average endpoint titer is shown with error bars represented as standard error. (**C**) Individual immune mouse sera (10 mice per cohort) evaluated in a long-range time course study demonstrated the highest peak ELISA titer to rCSP with the GLA-LSQ formulation ∼8 weeks after the final immunization with a 2.5 µg of rCSP administered per dose for each formulation tested. Average titer at OD = 1 is shown with error bars represented as standard error. Note that a standard scale (rather than log scale) is shown for the y-axis.

While inclusion of the disaggregant (urea) and a reducing agent in process buffers resulted in low levels of rCSP multimerization, ultimately, a third column (utilizing hydrophobic interaction chromatography) was added to the purification scheme as an additional polishing step and to further reduce the level of rCSP dimer in the final product. The successful expression and production of rCSP allowed us to further advance product development by moving forward in search of appropriate formulations suitable for clinical testing. Along with two common alum-based adjuvant systems, we also selected two TLR4 agonist-based adjuvants, GLA-SE and GLA-LSQ, for testing. Both formulations consist of a highly pure [30], synthetic hexaacylated monophosphorylated lipid A-like structure that has been shown to be an effective adjuvant for vaccine candidates against other infectious diseases [31], [32], [33]. Another important consideration for optimal adjuvant effectiveness is to improve formulation by using appropriate systems to increase uptake by cells, particularly antigen-presenting cells, and to provide sustained release of the antigen. In the case of GLA-SE, the GLA is formulated into a squalene-in-water nanoemulsion. In contrast, GLA-LSQ is a nanoliposomal formulation that includes QS21, a saponin. Each adjuvant formulation was mixed with rCSP prior to immunization. Protein formulated in incomplete Freund's adjuvant was also included in several experiments to bridge these data with the previously detailed down-selection data. Immunization with rCSP in the context of GLA-SE, GLA-LSQ, and incomplete Freund's adjuvant formulations resulted in similar immunogenicity of the different sera when evaluated by ELISA and IFA, whereas the titers of the sera obtained from mice immunized with both alum-based adjuvants were considerably lower (Figure 4a and 4b). ELISA titer was calculated at OD = 1.0; sera from mice injected with adjuvant only (each adjuvant was tested) did not achieve ODs of 1.0 by ELISA (ODs were <0.15 with these sera pools). The pattern of *Pb*-CS(*Pf*) sporozoite surface fluorescence with sera from all formulations evaluated in IFA was characteristic of reactivity to CSP and similar to that shown in Figure 3b (data not shown).

To further investigate whether the vaccine-induced immune responses are sustainable, the longevity of the antigen-specific humoral immune response to each of the rCSP formulations was measured in a time-course study throughout the entire injection schedule and for several months following the final injection (Figure 4c). Peak antibody response was seen 2 or 8 weeks after the final injection, depending on the formulation, with the GLA-LSQ formulation demonstrating the highest antibody response overall, followed by GLA-SE and then the alum-based formulations. Sera from mice injected with adjuvant-only did not achieve an OD of 1.0 by ELISA (ODs were <0.15 with these sera). The overall titers achieved with the GLA-SE and GLA-LSQ formulations were significantly higher compared to the alum-based formulations. In addition, the overall titers achieved with the GLA-LSQ formulation were significantly higher compared to the GLA-SE formulation (repeated measures ANOVA and post hoc comparisons). At the final collection time point (approximately 22 weeks after the final immunization), the titers achieved with the GLA-LSQ continued to be significantly higher compared to the GLA-SE and alhydrogel formulations (p values  = 0.003 and 0.00003, respectively using significant comparisons [Tukey] from a one-way ANOVA).

**Figure 5:**
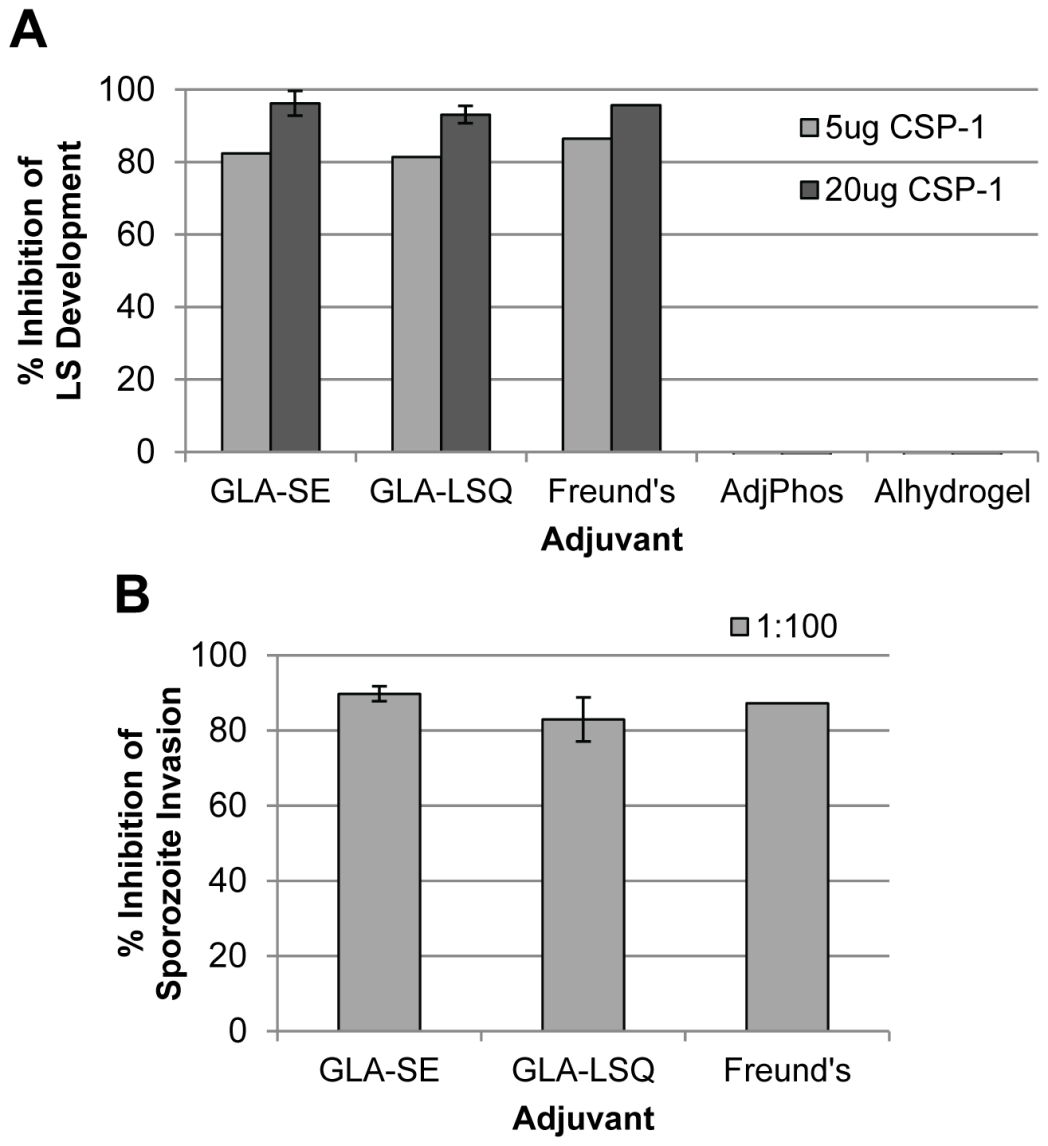
ISI and Transgenic Parasite Protection Comparisons of rCSP Adjuvant Formulations. Seven C57BL/6 mice per cohort were immunized 3x with 5 µg or 20 µg of rCSP (CSP-1) formulated in different adjuvants. Sera were collected 2 weeks after the last immunization and pooled for analysis. Mice were challenged with *Pb*-CS(*Pf*) parasites 3 weeks after the last immunization. (**A**) Percent inhibition of LS parasite development in rCSP-immunized mice versus naïve mice is shown upon *Pb*-CS(*Pf*) sporozoites challenge. A statistically significant reduction of LS parasites in livers of mice immunized with the GLA-SE or GLA-LSQ formulations was seen compared to the level of LS parasites in livers of naïve and adjuvant-only control mice. However, no reduction of LS parasites was seen with alum-based adjuvants. GLA-SE and GLA-LSQ formulations with 20 µg rCSP were assessed in multiple independent studies (5–7 mice per cohort for each study); average percent inhibition is shown with error bars represented as standard error. (**B**) Sera from mice immunized with rCSP in different GLA formulations were diluted 1∶100 or 1∶500 and assayed for the ability to block *Pf* sporozoite infection of hepatocytes in an ISI assay. Percent inhibition is shown relative to a naïve sera control. Sera from GLA-SE adjuvanted mice demonstrated higher ISI activity compared to sera from those adjuvanted with GLA-LSQ. Where multiple independent experiments were performed, average percent inhibition is shown with error bars represented as standard error.

The ability of the GLA-SE and GLA-LSQ formulations to induce protective immunity was also comparable to that of the incomplete Freund's adjuvant formulation. Upon challenge of the immunized mice with *Pb*-CS(*Pf*) sporozoites, all three formulations resulted in significant inhibition of LS parasite development (compared to the naïve and adjuvant-only control mice), achieving >80% inhibition when administered as a 5 µg dose of rCSP and >90% inhibition when administered as a 20 µg dose of rCSP with these adjuvants (Figure 5a). Likewise, an ISI assay showed that a 1∶100 dilution of the sera obtained from mice immunized with rCSP in the context of either GLA formulation resulted in a comparable reduction in sporozoite invasion to that observed with sera from mice immunized with rCSP emulsified in incomplete Freund's adjuvant (Figure 5b). Since neither of the rCSP alum-based formulations showed effectiveness in the LS inhibition assay, the immune sera was not tested by ISI, and these formulations were not considered for further testing.

### Sterile Protection Induced by Adjuvanted rCSP in Mice

While assessment of the amounts of parasite-specific rRNA in the liver by a real-time qRT-PCR translates to the percentage reduction of liver parasite load and provides a quantitative measure of protective efficacy, we also wanted to measure the level of sterile protection achieved upon vaccination with our lead candidate antigen-adjuvant combinations by monitoring the presence or absence of blood stage parasites following sporozoite challenge. Therefore, mice were immunized three times with 25 µg of rCSP formulated in either GLA-SE or GLA-LSQ and challenged 2 weeks after the last immunization. The presence of parasites in the blood was monitored by daily blood smear; sterile protection was determined as lack of parasites in thin blood smears (Table 1). The GLA-SE formulation exhibited slightly higher sterile protection (50%) as compared to the GLA-LSQ formulation (40%). No protection was seen in the adjuvant control or naïve mouse groups.

**Table 1:** Sterile Protection with rCSP.

Immunization	No. Infected/No. Challenged Mice	Sterile Protection (%)
rCSP (25 µg) + GLA-SE (5 µg)	5/10	50
rCSP (25 µg) + GLA-LSQ (5 µg)	6/10	40
GLA-SE (5 µg)	10/10	0
None (naïve)	10/10	0

BALB/c mice were immunized 3x with 25 µg of rCSP formulated in GLA-SE or GLA-LSQ, followed by challenge with Pb-CS(Pf) parasites 2 weeks after the last immunization. Parasitemia was monitored by Giemsa-stained blood smears daily from 7 to 11 days post challenge. Animals with no detectable parasitemia on all days monitored were considered sterilely protected.

### Cell-Mediated Immunity Elicited by Adjuvanted rCSP

**Figure 6:**
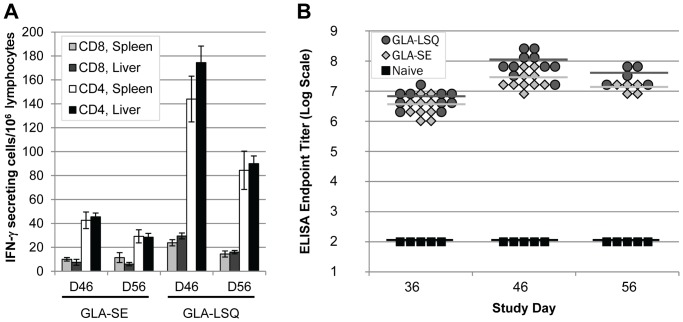
CSP-specific T-cell Response in Mice. Ten BALB/c mice (MHC haplotype H-2^d^) per cohort were immunized 3x with 25 µg of rCSP formulated in GLA-SE or GLA-LSQ. Lymphocytes (harvested from spleens and livers) were collected 10 and 20 days after the final immunization (i.e., study days 46 and 56, respectively) from 5 mice per cohort at each time point. Sera were collected on the day of the final immunization (before injection) and at 10 and 20 days post final immunization (i.e., study days 36, 46, and 56, respectively). No more than 2 IFN-γ producing cells/10^6^ lymphocytes were counted with cells harvested from mice injected adjuvant only (data not shown). (**A**) ELISpot assay was performed using either a H-2K^d^-restricted CD8 epitope containing peptide located in the N-terminus of the CSP, or a I-A^d^-restricted CD4 epitope containing peptide located in the C-terminus of the CSP for in vitro re-stimulation. Average numbers of IFN-γ producing cells/10^6^ lymphocytes are shown with error bars representing standard error of the mean. (**B**) Endpoint ELISA titers of sera collected from the same mice used for T-cell response assessments demonstrated high level of humoral responses (using rCSP to coat microtiter plates) induced in mice immunized with rCSP in both the GLA-SE and GLA-LSQ formulations.

Although the humoral response to the full-length rCSP is encouraging, the long-term effectiveness of a malaria vaccine may also require activation of the cell-mediated immune response. The level of cell-mediated immunity induced by the vaccine candidates was measured by ELISpot. Briefly, liver and spleen from immunized BALB/c mice (MHC haplotype H-2^d^) were harvested on study days 46 and 56, and lymphocytes were re-stimulated with peptides containing either an H-2A^d^ (I-A^d^)-restricted CD4 epitope (EYLNKIQNSLSTEWSPCSVT), which is located in the C-terminal region of CSP, or an H-2K^d^-restricted CD8 epitope (NYDNAGTNL), which is located in the N-terminal region of CSP. The relative number of epitope-specific, IFN-γ-secreting T-cells among lymphocytes of immunized mice were determined, as previously described [34]. Results showed that re-stimulation with the CD4 epitope-containing peptide produced a much stronger T-cell response than that of the CD8 epitope-containing peptide. Nevertheless, lymphocytes harvested from mice immunized with rCSP formulated with GLA-LSQ demonstrated higher responses to re-stimulation compared to those from mice immunized with rCSP formulated in GLA-SE (Figure 6a). A statistically significant difference in splenocytes harvested from mice immunized with the two formulations was seen with re-stimulation using both the CD4 peptide (p = 0.007 for day 46 and p = 0.015 for day 56) and CD8 peptide (p = 0.008 for day 46). In a similar experiment, three separate pools of overlapping peptides (15-mers) comprising the N-terminal region, central repeat region, or the C-terminal region of CSP were also used for re-stimulation. Similar results, albeit with slightly lower levels of IFN-γ secreting T lymphocytes, were found with the N-terminal and C-terminal peptide pools as compared to the re-stimulation with CD8 and CD4 epitope-containing peptides, respectively. As anticipated, no response above baseline was seen when the lymphocytes were re-stimulated with the central repeat peptide pool (data not shown), which has a putative H-2A^b^ (I-A^b^)-restricted CD4 epitope but no putative H-2^d^ epitope.

An ELISA was performed with sera collected from the same mice used for ELISpot assays, with rCSP as an antigen. We confirmed that rCSP formulated in either of the GLA adjuvants induced a strong CSP-specific antibody response as compared to either naïve animals (Figure 6b) or mice immunized with adjuvants only (data not shown). Consistent with our previous ELISA results, in which the antibody response was measured to either the CSP central repeat region or to the recombinant protein (see Figure 4), sera titers in mice immunized with the rCSP GLA-LSQ formulation trended higher than in mice immunized with the rCSP GLA-SE formulation with a statistically significant difference seen on day 46 (p = 0.089, p = 0.001, and p = 0.056 on study days 36, 46, and 56, respectively).

### Heterologous Prime-Boost Strategies Using rCSP and Ad35CS

Prime-boost vaccination regimens are standard for many licensed products; however, the concept of priming and boosting with different vaccine delivery methods (i.e., viral vectored and recombinant protein) while utilizing the same antigen (heterologous prime-boost) is a more recent strategy that has shown promise for improving the breadth of immunogenicity and protection [18], [35], [36]. For CSP-based vaccines, preclinical studies have demonstrated the effectiveness of this strategy [37], [38]. In particular, adenovirus has been shown to be an excellent delivery vehicle for induction of cellular-mediated immunity [39], [40], [41], [42] and has been used in other heterologous prime-boost studies [43]. Therefore, a heterologous prime-boost strategy was evaluated using rCSP formulated with GLA-SE and Ad35CS, a replication-deficient, recombinant human adenovirus serotype 35 vector expressing the full-length PfCSP, previously demonstrated as safe in Phase 1 clinical trials [44], [45], [46]. GLA-SE was selected as the adjuvant for rCSP in these studies since this adjuvant has previously been used in the clinic, whereas GLA-LSQ has not yet been tested in humans. While the reported data generally point to priming with a viral or DNA vectored construct, followed by a boost with the recombinant protein (i.e., boosting T-cell responses followed by B-cell responses) as a successful strategy [47], we tested all of the permutations using a 3-dose regimen so as not to bias the results (Table 2).

**Table 2:** rCSP/Ad35CS Heterologous Prime-Boost Regimens.

Group	Injection Type and Day (D)			Regimen Abbreviation
	D = 0	D = 14	D = 36	
1	Ad35CS	Ad35CS	Ad35CS	Ad35CS 3x
2	GLA-SE+rCSP	GLA-SE+rCSP	GLA-SE+rCSP	GLA-SE 3x
3	GLA-SE+rCSP	Ad35CS	Ad35CS	GLA-SE 1x/Ad35CS 2x
4	GLA-SE+rCSP	GLA-SE+rCSP	Ad35CS	GLA-SE 2x/Ad35CS 1x
5	Ad35CS	GLA-SE+rCSP	GLA-SE+rCSP	Ad35CS 1x/GLA-SE 2x
6	Ad35CS	Ad35CS	GLA-SE+rCSP	Ad35CS 2x/GLA-SE 1x
7	Naïve			

C57BL/6 mice were immunized with rCSP formulated in GLA-SE or Ad35CS according to the regimens shown.

**Figure 7:**
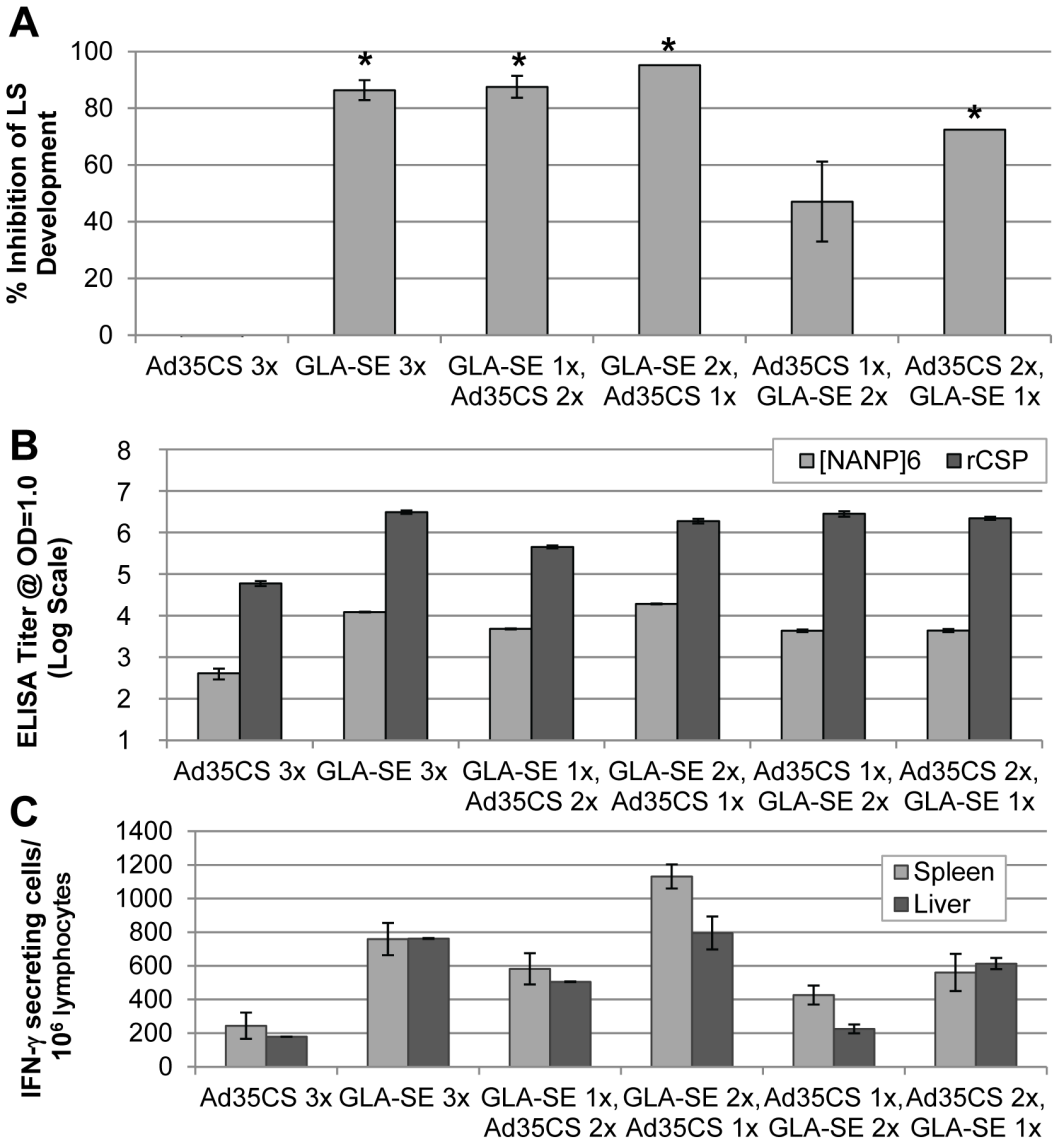
Activity of Prime-Boost Regimens in Mice. C57BL/6 mice (5–7 per cohort) were immunized 3x with 20 µg of rCSP formulated in GLA-SE, 1–2×10^10^ v.p. of Ad35CS, or a combination of the two. Spleens and livers were collected 10 days after the last immunization; sera were collected 10 or 14 days after the last immunization. To assess reduction in parasite load in the liver or sterile protection, mice were challenged with *Pb*-CS(*Pf*) parasites 3 weeks after the last immunization. (**A**) Percent inhibition of LS parasite development in immunized mice challenged with *Pb*-CS(*Pf*) sporozoites normalized to naïve mice received the challenge, and percent sterile protection in immunized BALB/c mice upon challenge with a fewer number of Pb-CS(Pf) sporozoites are shown. A statistically significant reduction of LS parasites in the livers of mice immunized with all regimens, except Ad35CS 3x and Ad35CS (1x), GLA-SE (2x), was seen upon sporozoites challenge, compared to the level of LS parasites in the livers of negative control mice following sporozoites challenge, based on the mean parasite's 18s rRNA copy number (as noted by asterisks). Three regimens were assessed in two independent studies (GLA-SE 3x; GLA-SE 1x, Ad35CS 2x; and Ad35CS 1x, GLA-SE 2x); for these regimens, average percent inhibition is shown with error bars represented as standard error. Statistical significance with the GLA-SE 3x; GLA-SE 1x, Ad35CS 2x was seen in both studies. (**B**) ELISA was used to evaluate the level of humoral response in immunized mice to the repeat region of CSP with pooled mouse sera and to whole rCSP with individual mouse sera. Titer was assessed at OD = 1.0 based on 4-parameter logistic curve fits. Average titers are shown with error bars representing standard error of the mean. (**C**) The level of T-cell response was determined via ELISpot using lymphocytes isolated from the spleens and livers of immunized mice (5 mice per cohort were used for this study) and overlapping peptides (15-mers encompassing the CSP repeat region) for in vitro restimulation. Average numbers of IFN-γ producing cells/10^6^ lymphocytes are shown with error bars representing standard error of the mean. No more than 5 IFN-γ producing cells/10^6^ lymphocytes were counted with cells harvested from naïve mice (data not shown).

Efficacy of the different prime-boost regimens was evaluated via challenge studies in C57BL/6 mice (Figure 7A). The GLA-SE 3x, GLA-SE 1x/Ad35CS 2x, GLA-SE 2x/Ad35CS 1x, and Ad35CS 2x/GLA-SE 1x regimens all resulted in significant decrease in liver parasite load compared to naïve mice. However, contrary to the previously established consensus, the greatest inhibition (>85%) was seen in regimens where rCSP + GLA-SE was administered first (GLA-SE 3x; GLA-SE 1x/Ad35CS 2x; and GLA-SE 2x/Ad35CS 1x). No reduction in liver parasite load was found in mice immunized with 3 injections of Ad35CS as compared to the naïve control group.

Humoral response was measured in the sera of immunized mice via ELISA, using either a repeat region peptide, [NANP]_6_, or rCSP as an antigen (Figure 7B). Sera titers (calculated at OD = 1) assessed with either antigen were similar across the different regimens except for animals immunized with the Ad35CS 3x regimen, for which titer was comparatively lower.

T-cell response was evaluated via ELISpot by harvesting lymphocytes (from both spleen and liver) 10 days post final immunization. Cells were re-stimulated using overlapping peptides (15-mers) comprising full-length CSP and divided into several pools encompassing specific regions of CSP (N-terminal, repeat, and C-terminal regions). Note that as C57BL/6 mice (H-2^b^) were used for this study, the H-2A^d^ (I-A^d^)- and H-2K^d^-restricted CD4 and CD8 peptides used in the previous study with BALB/c (H-2^d^) were not appropriate for re-stimulation, and, as expected, no activity was seen with either the N-terminal or C-terminal peptide pools (data not shown). However, positive responses were seen with lymphocytes harvested from both the spleen and liver re-stimulated with the repeat region peptide pool, likely resulting from the putative H-2A^d^ (I-A^d^)-restricted CD4 epitope present within the repeat region (Figure 7C). Striking differences in the levels of T-cell response were seen with the different regimens. Interestingly, the levels of T-cell response induced by different immunization regimens appear to trend with the levels of reduction in the liver parasite load in a challenge study (Figure 7A). Specifically, cells from mice immunized with the GLA-SE 3x; GLA-SE 1x/Ad35CS 2x; GLA-SE 2x/Ad35CS 1x; and Ad35CS 2x/GLA-SE 1x regimens showed the highest cellular responses, and those from mice immunized with the Ad35CS 3x and Ad35CS 1x/GLA-SE 2x regimens showed lower cellular response, comparatively. A statistically significant increase in cellular response was seen in splenocytes harvested from mice immunized with the GLA-SE 2x/Ad35CS 1x regimen as compared to all other regimens tested (p≤0.0159).

Altogether, the results from our heterologous prime-boost experiments indicate that although the vaccination with Ad35CS construct alone (Ad35CS 3x) is not very effective in the mouse model, a single dose of Ad35CS vaccination, following two doses of rCSP-GLA-SE (GLA-SE 2x/AD35CS 1x), appears to modestly improve the T-cell response over the rCSP-GLA-SE alone (GLA-SE 3x). Surprisingly, reversing the order of the prime-boost regimen - a single dose of Ad35CS followed by two rCSP-GLA-SE boosters (Ad35CS 1x/GLA-SE 2x) - markedly reduces the ability to elicit T-cell response and ultimately anti-malarial protection. These observations not only support the use of a heterologous prime-boost strategy as an effective vaccination approach but also highlight the importance in the order of delivery. Therefore, further studies are required to determine the optimal strategy to achieve the desired protection in the clinical setting.

## Discussion

In this study, manufacture of a full-length recombinant CSP was accomplished using a *P. fluorescens* expression system. The advantages of this expression system include the obviation of the need for antibiotics as it exerted positive selective pressure on resulting strains, and the simplification of the downstream processing scheme due to rCSP being secreted into the periplasmic space of the down-selected *P. fluorescens* strains. Significant challenges for rCSP expression were overcome through selection of a *P. fluorescens* expression strain with a specific protease deletion that results in improved levels of full length rCSP (strain CSP-1), implementation of steps to rapidly separate rCSP from any residual endogenous proteases that may progressively truncated the N-terminus of the protein, and suppression of rCSP aggregation during the purification process (presumably due to the presence of an unpaired N-terminal cysteine residue) by freezing the lysate, which was found to be a preferred step prior to initiating the entrained chromatographic purification process, as well as by inclusion of a disaggregate (urea) and a reducing agent in all load and process buffers. Although a 2-column purification process was sufficient to produce protein for analytical and biological activity screening, a 3-column purification process was ultimately implemented to reduce the level of rCSP dimer in the final product. Additionally, a suite of analytical assays were developed, including RP-HPLC, SEC-HPLC, SDS-PAGE, peptide mapping, and capillary iso-electric focusing, to biophysically monitor and characterize integrity of the molecule during in-process manufacturing, release, and long-term storage stability monitoring that will be used for clinical material development using the process described herein. The fermentation and purification processes described in this manuscript have been scaled up to accommodate manufacture at the 30 L scale. Therefore, to our knowledge, this is the first description of a process for generating clinical-grade full-length recombinant CSP that is suitable for economical large-scale production, clinical evaluation, and advanced product development.

To demonstrate that rCSP produced in the *P. fluorescens* expression system could be a potent immunogenic antigen against malaria infection in mice, we first measured immunogenic activity with a panel of biological assays upon rCSP vaccination. Humoral immune responses in mice were assessed via ELISA and IFA. An ISI assay was used to quantify the ability of functional antibodies to inhibit *Pf* sporozoite invasion of hepatocytes. Finally, protective efficacy of the rCSP-elicited immune response was determined by assessing the degree of liver stage parasite development upon challenge with *Pb*-CS(*Pf*) sporozoites in a mouse model. Upon vaccination, all three initial strains of rCSP elicited high levels of antibodies reactive against the repeat region of the CSP, but more importantly the antibodies raised by rCSP could recognize the native conformation of the protein present on the surface of the parasite. Among the 3 strains tested, one strain (CSP-1) induced a higher trend in ISI activity as compared to the other two strains. Intriguingly, while the titers of antibodies against the central repeat region of the CSP did not vary amongst the three strains, there was a marked decrease in biological activity by the CSP-2 strain derived protein. This is likely because the CSP-2 strain demonstrated the most N-terminally clipped protein (54%) and greatest number of amino acids clipped from the N-terminus (up to 17) as compared to the other strains. Not only did this negatively impact efficacy of the CSP-2 expression product but also may have changed the immune response to the protein such that a less efficacious response was generated. This may correlate with the fact that the central repeat region is the immunodominant B-cell domain and has been implicated as an immunogenic “decoy” in an immune evasion strategy of the parasite [48]. These observations may be of biological importance because the CSP N-terminus contains a proteolytic processing site that is cleaved prior to successful parasite invasion of the liver [10], [49]. Indeed, we have identified an N-terminus mAb that binds near or on the cleavage site, and have shown through passive transfer studies that this mAb is able to block the invasion (>90%) of malaria parasites in vivo (data not shown). Therefore, our data further bolsters the rationale for the development of full-length CSP vaccine candidate that includes the N-terminus of the protein.

CSP-1 was selected as the strain to move forward for further development of GMP-grade rCSP, and we proceeded with the selection of an appropriate adjuvant to take forward to the clinic. We chose two adjuvants containing a TLR4 agonist (GLA-SE and GLA-LSQ) and two alum-based adjuvants (alhydrogel and adjuphos) to combine with rCSP for these studies. CSP-specific antibody responses were augmented in the presence of TLR4 agonist-based adjuvants, but not with the two alum-based products. Accordingly, the TLR4 agonist-based adjuvants, but not the alum-based adjuvants, showed good reduction of liver parasite load at a 5 µg dose of rCSP (80%–90%) with a slight improvement at the higher 20 µg dose of rCSP (90%–99%). Furthermore, sera from mice immunized with the GLA formulations were capable of inhibiting *Pf* sporozoite invasion into human hepatocytes in vitro, as measured by an ISI assay. It is important to note that the ISI assay measures the ability of anti-CSP antibodies to neutralize sporozoite invasion; however, it has not yet been clearly established with regards to the mechanism of the inhibitory action by the antibodies. Interestingly, the degree of protection (reduction in parasite liver load) seen with alum-formulated rCSP correlates with the low serum titers in immunized mice, which is consistent with field study reports showing a correlation between anti-CSP antibody titer and degree of protection from *P. falciparum* infection among people living in endemic areas. However, the relationship between anti-CSP antibody titer and efficacy of RTS, S vaccine is more complicated in that a correlation was seen in naïve adults and young children of some endemic regions but not in other African populations [50], [51]. Recent analysis of the RTS, S studies has revealed a synergistic effect between the antibody response and cellular response, particularly of CD4+ T cells, to CSP, leading to the modest protection now seen in their Phase 3 trials [52]. These studies underscore the need to elicit both arms of robust T-cell and B-cell responses in order to effectively protect from malaria infection.

Importantly, GLA-SE has been shown to elicit robust T-cell and B-cell responses induced by several malaria antigens including CelTOS and other forms of recombinant CSP [27], [53]. This is the first reported study using GLA-LSQ, and we observed both an increased antibody response as well as an enhanced CSP-specific CD4+ T cell response rather than CD8+ T cell response, by the rCSP GLA-LSQ formulation as compared to the rCSP GLA-SE formulation. Note that the GLA-LSQ adjuvant is currently under development for use in clinical trials. Two completed clinical studies of pre-erythrocytic vaccines containing GLA-SE have demonstrated that GLA-SE is safe and well-tolerated [54], [55], and there are multiple ongoing clinical studies using GLA-SE as adjuvant for malaria and other vaccines. In view of its pre-clinical performance with the rCSP and its safety profile, we plan to proceed with this adjuvant in combination with rCSP in the initial clinical trial.

Although its precise mechanism of action is still unknown, saponin clearly augments antibody and T-cell responses in synergy with TLR4 ligands. Nanoparticle adjuvant formulations similar to GLA-LSQ or GLA-SE are amenable to uptake by antigen presenting cells (APCs), which specialize in the phagocytosis of invading particulate pathogens and foreign particles. Additionally, delivery of antigens and adjuvants as complex particles can provide a sustained release mechanism, allowing for multimeric presentation to TLRs and APCs, and providing a delivery vehicle for otherwise insoluble components. Our data suggest different immunological profiles are elicited for GLA-SE and GLA-LSQ in combination with rCSP when administered to mice, which may also occur in humans and lead to different clinical outcomes for the two adjuvants. We are currently proceeding with the GMP process development of the GLA-LSQ so that we can measure its safety and immunogenicity in combination with rCSP in the clinic as well.

While our efforts to choose an adjuvant involve the enhancement of cell-mediated immunity targeted against the liver stage of parasite, we are well aware that immunization with recombinant proteins does not typically promote optimal T-cell response. Indeed in our initial study with BALB/c mice, we were able to enhance CSP-specific CD4+ T-cell response only modestly over baseline levels using rCSP formulated in GLA adjuvants. Therefore, we sought to test the effects of a heterologous prime-boost strategy in a series of studies using rCSP adjuvanted with GLA-SE in combination with an adenovirus-based CSP vaccine, since adenoviral vectors are known to induce a potent T-cell response [39], [40], [41]. Our results showed that the regimen where GLA-SE + rCSP was administered prior to Ad35CS demonstrated the most potent efficacy and resulted in the highest reduction in the development of liver stage parasites among the regimens tested. Additionally, this immunization regimen induced strong responses mediated by both B-cell and T-cell compartments, as assessed by measuring the humoral and cellular responses. There is precedent for heterologous prime-boost strategies to improve the magnitude, specificity or breadth of the host immune response [43]; however, it is typically seen as the viral vectored or DNA as prime followed by a recombinant protein boost [18], [47]. Interestingly, there have been studies with recombinant CSP and adenovirus-delivered CSP that follow this dogma [38], [43], [56]. Of note is a prime boost study conducted with adenovirus and GLA-SE adjuvanted recombinant protein vaccine candidates against *Mycobacterium tuberculosis* wherein long lived protection in mice was observed only when the adjuvanted protein vaccine candidate was administered prior to the adenovirus vaccine candidate [57]. Our results too suggest that administration of recombinant protein as prime followed by a recombinant adenovirus expressing the same protein as boost may improve efficacy of the vaccine, underlining the need to test all possible regimen combinations in order to achieve optimal performance. We are currently exploring the use of VLPs as a means to deliver critical epitopes of the CSP using several different technology platforms, which may be able to efficiently engage and induce potent “protective” anti-plasmodial immune responses mediated by both antibodies and T cells.

## Materials and Methods

### CSP Cloning, Strain Selection, Production, and Purification

The full-length CSP gene sequence, encoding *P. falciparum* 3D7 isolate (CAB38998) amino acids 21–382, was optimized for expression in *P. fluorescens* (DNA2.0; Menlo Park, California) and cloned into sixteen expression plasmids (Pfenex, Inc.; San Diego, California). Twenty *P. fluorescens* host strains were electroporated with each plasmid in a 96-well format, resulting in 320 expression strains [58]. Small-scale fermentation was performed with protein expression analysis by SDS-PAGE and western blotting to determine expression product levels. An anti-PfCSP conformational monoclonal antibody (mAb 4C2) used in screening was kindly provided by the Laboratory of Malaria Immunology and Vaccinology (NIAID) [28]. Bioreactor fermentations at a 1 L scale were performed for down-selected strains. Dissolved oxygen was maintained at a positive level in the liquid culture by regulating the sparging air flow, oxygen flow, and agitation rates after inoculation. The pH was controlled at the desired set-point through the addition of aqueous ammonia. The fed-batch high cell density fermentation process was divided into an initial growth phase and gene expression phase in which IPTG was added to initiate recombinant gene expression and the expression phase of the fermentation was allowed to proceed for 24 hours. Frozen cell pastes thawed at room temperature were suspended in a Tris-urea buffer and lysed by microfluidization at 15,000 psi. Lysates were clarified by continuous flow centrifugation, filtered (0.2 µm), and frozen at −70°C. Just prior to downstream purification, frozen lyates (thawed in a water bath at room temperature) were batch centrifuged and filtered (0.2 µm). 2 M urea was used in all load and process buffers to suppress protein aggregation. A two-step purification process was developed using Q-Sepharose (GE Healthcare) anion-exchange chromatography and hydrophobic interaction chromatography (Butyl 650S, Tosoh Bioscience LLC). The final eluate was exchanged into 1X PBS buffer by dialysis (regenerated cellulose, 10K MWCO; Thermo Scientific), followed by concentration with centrifugal ultrafiltration membrane filters (regenerated cellulose, 10K MWCO, Millipore).

### CSP Analytical Characterization

Purity assessments of rCSP were made via SE chromatography and RP chromatography. SE chromatography was performed using a TSKgel G3000SWXL column (Tosoh Bioscience LLC) equipped to an Agilent 1100 HPLC system. PBS was used as the mobile phase with a 0.5 mL/minute flow rate and 50–100 µL injection volume; absorbance was monitored at 280 nm. RP-HPLC was performed on an Agilent 1100 HPLC system using a Jupiter C4 column (Phenomenex). Mobile phase A contained 0.1% trifluoreacetic acid (TFA) in water (v/v); solvent B contained 0.1% TFA in acetonitrile (v/v). Gradient conditions were 22%–32% solvent B with a 1 mL/minute flow rate and 30–60 µL injection volume (samples were diluted in PBS); absorbance was monitored at 214 nm and 280 nm. Integrity and identity of each rCSP were determined by assessment of intact mass and N-terminal sequencing. Intact mass was assessed with liquid chromatography coupled to mass spectrometry. Briefly, reduced samples were analyzed using an Agilent 1100 HPLC system coupled to a Q-T micro mass spectrometer (Waters) with an electrospray interface. A C_8_ column (Zorbax 5 µm, Agilent) was used for separation and UV absorbance collected from 180–500 nm. UV chromatograms and MS total ion current chromatograms were generated and spectra deconvoluted using MaxEnt 1 (Waters) scanning for a molecular weight range of 30,000–50,000 at a resolution of 1 Da per channel. For N-terminal sequencing (American International Biotechnology, LLC; Richmond, Virginia), samples were loaded onto a PVDF sequencing membrane and subjected to 36 cycles of Edman degradation using a Procise Protein Sequencer (Applied Biosystems, Inc.). After each cycle, amino acids were separated and quantified using the integrated chromatographic system and the resultant sequence(s) compared to the expected *P. falciparum* 3D7 CS protein sequence.

### Mice

Animal studies were conducted at The Rockefeller University (OLAW number A3081-01), Johns Hopkins University (OLAW number A3272-01), and Molecular Diagnostic Services, Inc. (OLAW number A4202-01). Six- to 8-week old female C57BL/6 mice were purchased from NCI (Frederick, Maryland) or Taconic (Germantown, New York). Six- to 8-week old female BALB/c mice were purchased from Taconic. All mice were maintained under standard conditions by the facility conducting the study, and all animal studies were approved by the respective Institutional Animal Care and Use Committee.

### Immunizations

Mice were injected via the intramuscular (IM) route on study days 0, 14, and 36 with 2.5 µg, 5 µg, 20 µg, or 25 µg of rCSP formulated with an adjuvant. Several adjuvants were utilized including 50% Alhydrogel (Brenntag), 50% Adjuphos (Brenntag), 5 µg GLA-SE (Infectious Disease Research Institute) and 5 µg GLA-LSQ (Infectious Disease Research Institute) in combination with antigen. In addition, incomplete Freund's adjuvant (Sigma) was used as a positive control. Naïve mice and/or mice immunized with adjuvant alone served as negative controls. For some experiments, mice immunized via the IM route with a recombinant adenovirus expressing PfCSP were included – 2×10^10^ AdCS [34] or 1–2×10^10^ Ad35CS (produced by Crucell) [40] viral particles (v.p.) per dose.

### Humoral Response in Mice

Immunogenicity of C57BL/6 pooled mouse sera (collected on study day 50) to the PfCSP repeat region was evaluated via ELISA using a [NANP]_6_ peptide coated on the ELISA plates as described in Kastenmuller et al., 2013 [27]. Immunogenicity of individual mouse serum to the full length rCSP was also evaluated by ELISA using a method similar to that used for the extended time course study detailed below. Titer at an optical density (OD) of 1.0 was determined based on a 4-parameter logistic regression model with the calibFit package [59] for R [60]. Statistical significance for assessments of individual mouse sera was calculated using Mann-Whitney U.

An immunogenicity study of sera collected from immunized C57BL/6 mice over an extended period of time was performed by Molecular Diagnostic Services, Inc. (San Diego, CA). Individual mouse sera collected on study days -2 (pre-bleed), 11, 34, 50, 89, 140, and 189 were tested by ELISA. Briefly, Maxisorp ELISA plates were coated with 100 or 200 ng/well of rCSP, incubated at 4°C for overnight, washed and blocked with PBS containing 1% BSA (PBS-1% BSA). Plates were washed again and incubated with either 2- or 3-fold serial dilutions of serum starting at a 1∶2,000 dilution. After 1-hour incubation at room temperature (RT), plates were washed and incubated with an HRP-labeled goat anti-mouse secondary antibody, followed by addition of 3, 3′, 5, 5′ tetramethyl benzidine (TMB) substrate and stop buffer. Finally, the plates were read at 450 nm by spectrophotometer and titers calculated either based on a 4-parameter curve fit at an OD of 1.0 or determined by the maximal dilution that gave a value of OD = 0.1 (endpoint titer). Statistical analysis of the extended time course study data was done by repeated measures ANOVA and post hoc comparisons to compare titers achieved for each formulation across the study, and significant comparisons (Tukey) from a one-way ANOVA to compare titers at the defined collection time points. For the analysis, data were log+1 transformed to better meet assumptions of ANOVA and adjustments made using corrections based on the Mauchly's Test for sphericity.

### Immunofluorescence Assay (IFA)

Reactivity of the sera collected from immunized mice to transgenic *P. berghei* sporozoites (*Pb*-CS(*Pf*) expressing the *P. falciparum* CSP repeat region and a portion of the N-terminal region including the cleavage site), kindly provided by Dr. Elizabeth Nardin [61], was tested by IFA. Briefly, IFA slides were first coated with the sporozoites and fixed with 0.05% glutaraldehyde and were incubated for 30 minutes with 3-fold serial dilutions of the sera. Slides were then washed with PBS-1% BSA, and a secondary antibody, Alexa Fluor 488-labeled F(ab')2 fragment of goat anti-mouse IgG (H+L), was added for 30 minutes. Finally, the slides were visualized with a fluorescence microscope (Nikon Eclipse 90i) and endpoint titers determined as the lowest serum dilution to give sporozoite surface fluorescence above negative sera control level.

### Inhibition of Sporozoite Invasion (ISI)

HepG2-A16 cells (a human hepatocyte line) were seeded on 8-chamber slides and grown to monolayer. Diluted pool serum samples followed by 25,000 *P. falciparum* (NF54) sporozoites per well at a final dilution of 1∶50, 1∶100, or 1∶500 were added to triplicate wells on the chamber slides. Cultures were incubated for 3 hours at 37°C, then washed 2X with PBS and fixed with 4% paraformaldehyde. The slides were then immuno-stained with mAb 2A10 (specific for CSP) to identify invaded sporozoites. The number of sporozoites, having invaded the hepatoma cells, was then determined by microscopic counting of the early-invaded forms using an epifluorescence microscope. Percent inhibition was calculated as mean sporozoites/well in negative control well minus the mean sporozoites/well in the test sample wells divided by the mean sporozoites/well in negative control wells multiplied by 100.

### T-cell Response in Mice

Liver and spleen from immunized BALB/c and C57BL/6 mice were harvested on study days 46 and 56, and lymphocytes were isolated from respective organ to determine the level of T-cell responses via an ELISpot assay. Individual spleens were analyzed; whereas 2–3 livers were pooled for the analysis. For the experiment performed using lymphocytes derived from BALB/c mice, peptides containing either an I-A^d^-restricted CD4 epitope (EYLNKIQNSLSTEWSPCSVT) located in the C-terminal region of the CSP and an H-2K^d^-restricted CD8 epitope (NYDNAGTNL) located in the N-terminal region of the CSP were used for in vitro restimulation. For the experiment conducted using lymphocytes derived from C57BL/6 mice, overlapping peptides (15-mers) encompassing the N-terminal, repeat region, and C-terminal regions of CSP were used for the restimulation. The relative number of epitope-specific, IFN-γ-secreting T cells among lymphocytes of immunized mice were then determined, as previously described in Shiratsuchi et al., 2010 [34]. Statistical significance was calculated using Mann-Whitney U.

### Sporozoite Challenge

For evaluation of the protective efficacy of the vaccine candidates, the amount of parasite load in the liver was determined upon challenging C57BL/6 mice by intravenous administration of 1×10^4^ transgenic *Pb*-CS(*Pf*) sporozoites. Briefly, 42 hours post challenge, mice were euthanized and the liver was excised from each mouse. Estimation of the amount of parasite-specific 18S rRNA in each liver was determined by quantitative RT-PCR assay, and percent inhibition of the parasite development in the liver was calculated as previously described [62]. Statistical significance was calculated using Mann-Whitney U.

For evaluation of sterile protection, BALB/c mice were immunized as described above, except the time interval between the second and third immunizations was 1 month. Mice were challenged 2 weeks after the third immunization by intravenous administration of 5000 transgenic *Pb*-CS(*Pf*) sporozoites. The presence of parasitemia was determined via microscopic evaluation of Giemsa-stained blood smears daily on days 7–11 post challenge.
